# Targeting of immune checkpoint regulator V-domain Ig suppressor of T-cell activation (VISTA) with ^89^Zr-labelled CI-8993

**DOI:** 10.1007/s00259-024-06854-z

**Published:** 2024-07-26

**Authors:** Ingrid Julienne Georgette Burvenich, Christian Werner Wichmann, Alexander Franklin McDonald, Nancy Guo, Angela Rigopoulos, Nhi Huynh, Mary Vail, Stacey Allen, Graeme Joseph O’Keefe, Fiona Elizabeth Scott, Raul Soikes, Steven Angelides, Reinhard von Roemeling, Andrew Mark Scott

**Affiliations:** 1 Tumour Targeting Laboratory, Olivia Newton-John Cancer Research Institute, Level 5 ONJ Centre, 145 Studley Road, Heidelberg, VIC 3084 Australia; 2 School of Cancer Medicine, La Trobe University, Melbourne, VIC Australia; 3 Curis Inc, Lexington, MA USA; 4 Department of Medicine, University of Melbourne, Melbourne, VIC Australia; 5 Department of Molecular Imaging and Therapy, Austin Health, Melbourne, VIC Australia

**Keywords:** CI-8993, VISTA, Zirconium-89, Theranostic, Immune checkpoint, PET

## Abstract

**Background:**

CI-8993 is a fully human IgG1κ monoclonal antibody (mAb) that binds specifically to immune checkpoint molecule VISTA (V-domain Ig suppressor of T-cell activation). Phase I safety has been established in patients with advanced cancer (NCT02671955). To determine the pharmacokinetics and biodistribution of CI-8993 in patients, we aimed to develop ^89^Zr-labelled CI-8993 and validate PET imaging and quantitation in preclinical models prior to a planned human bioimaging trial.

**Methods:**

CI-8993 and human isotype IgG1 control were conjugated to the metal ion chelator *p*-isothiocyanatobenzyl-desferrioxamine (Df). Quality of conjugates were assessed by SE-HPLC, SDS-PAGE, and FACS. After radiolabelling with zirconium-89 (^89^Zr), radioconjugates were assessed for radiochemical purity, immunoreactivity, antigen binding affinity, and serum stability in vitro. [^89^Zr]Zr-Df-CI-8993 alone (1 mg/kg, 4.6 MBq) or in combination with 30 mg/kg unlabelled CI-8993, as well as isotype control [^89^Zr]Zr-Df-IgG1 (1 mg/kg, 4.6 MBq) were assessed in human VISTA knock-in female (C57BL/6 N-Vsir^tm1.1(VSIR)Geno^, huVISTA KI) or control C57BL/6 mice bearing syngeneic MB49 bladder cancer tumours; and in BALB/c *nu*/*nu* mice bearing pancreatic Capan-2 tumours.

**Results:**

Stable constructs with an average chelator-to-antibody ratio of 1.81 were achieved. SDS-PAGE and SE-HPLC showed integrity of CI-8993 was maintained after conjugation; and ELISA indicated no impact of conjugation and radiolabelling on binding to human VISTA. PET imaging and biodistribution in MB49 tumour-bearing huVISTA KI female mice showed specific localisation of [^89^Zr]Zr-Df-CI-8993 to VISTA in spleen and tumour tissues expressing human VISTA. Specific tumour uptake was also demonstrated in Capan-2 xenografted BALB/c *nu*/*nu* mice.

**Conclusions:**

We radiolabelled and validated [^89^Zr]Zr-Df-CI-8993 for specific binding to huVISTA in vivo. Our results demonstrate that ^89^Zr-labelled CI-8993 is now suitable for targeting and imaging VISTA expression in human trials.

**Graphical Abstract:**

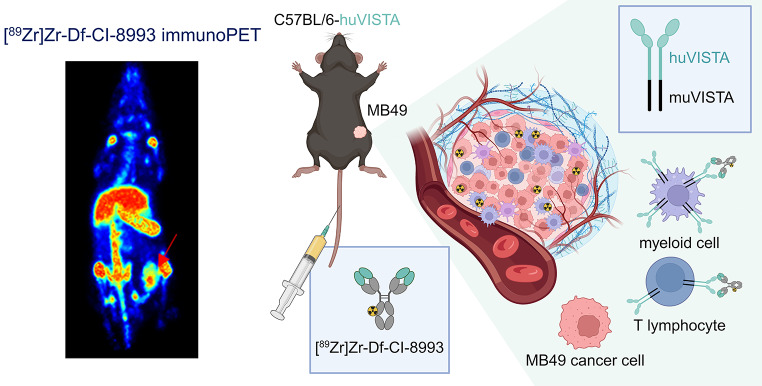

**Supplementary Information:**

The online version contains supplementary material available at 10.1007/s00259-024-06854-z.

## Introduction

Checkpoint inhibitors (ICI) such as anti-CTLA4 (ipilimumab), anti-PD1 (pembrolizumab, nivolumab, cemiplimab, dostarlimab, retifanilab), anti-PD-L1 (atezolizumab, avelumab, durvalumab), anti-LAG-3 (relatlimab) and anti-TIGIT (tiragolumab) antibodies have been effective in treating multiple tumour types [1]. However, not every patient benefits from these drugs and some patients experience severe immune-related adverse events [2] or develop drug resistance [3]. Biomarkers have been investigated to predict patient outcome and/or toxicities [4–7]. Excitingly, PET imaging has emerged as a tool that can potentially play an important role in managing checkpoint inhibitor development, via selecting patients that might benefit from immune checkpoint inhibitor therapy and/or by predicting if patients will develop severe toxicities [8–10].

V-domain immunoglobulin suppressor of T-cell activation (VISTA) is a member of the inhibitory B7 family of immune checkpoint proteins [11, 12]. VISTA expression is found on hematopoietic cells. It is constitutively and highly expressed on CD11b^+^ myeloid cells (neutrophils, monocytes, macrophages, and dendritic cells), and expressed at lower levels on naïve CD4^+^ and CD8^+^ T cells and regulatory T cells (Tregs) both in humans and mice. Unlike PD-1 and CTLA-4 which are expressed on activated T lymphocytes, VISTA is found on naïve T cells; and thought to be a negative regulator of T cell activation, keeping the T cells in a dormant state by blunting the production of T-cell cytokines and activation markers [13, 14]. In multiple murine cancer models, VISTA has been found at high expression levels on myeloid cells that infiltrate the tumours (myeloid-derived suppressor cells and T regulatory cells), suggesting VISTA plays a role in tumour immune cell evasion [15]. In combination with PD-L1 or CTLA-4 targeted therapy, anti-VISTA antibody therapy has shown tumour regression and synergistic effect in preclinical tumour models, suggesting it might help overcome immune check point inhibitor drug resistance [16–18].

CI-8993 is a fully human IgG1κ monoclonal antibody that binds specifically to VISTA [19]. The safety of CI-8993 has been established in a Phase I trial in patients with advanced cancer (NCT02671955). To assist determining the pharmacokinetics and biodistribution of CI-8993 in patients we aimed to develop zirconium-89 (^89^Zr)-labelled CI-8993 for PET imaging and biodistribution, and validate in preclinical models prior to a planned human trial.

## Materials and methods

### Reagents

CI-8993 (JNJ-61610588, Lot 15I28), a fully human IgG1 anti-human VISTA antibody was provided by Curis Inc. (Lexington, MA) in a formulation of 10 mM acetate buffer, 8.5% sucrose, 0.04% polysorbate 20, 20 µg/mL EDTA (pH 5.2). Control chimeric VISTA binding antibody VSTB124 (Lot 7947), comprises the same variable region as CI-8993, and a silent mouse IgG2a Fc that does not bind to Fc receptors and was provided by ImmuNext Inc (Lebanon, NH). An isotype control human IgG1 non-VISTA binding monoclonal antibody (mAb) was provided by ONJCRI.

### Cell culture

The murine bladder cancer cell line MB49 was obtained from EMD Millipore (SCC148; Temecula, CA). Capan-2 human pancreatic cancer cells, U-87 MG human glioblastoma cells and K-562 human lymphoblast cells were obtained from the American Type Culture Collection (ATCC, Manassas, MD, USA). All cell lines were cultured in DMEM (Invitrogen, Carlsbad, CA, USA) with 10% fetal calf serum and incubated at 37 °C with 5% CO_2_.

### Flow cytometry

VISTA antigen binding by anti-VISTA CI-8993 and human IgG1 control antibodies (20 µg/mL) was assessed using 1 × 10^6^ MB49 or Capan-2 cells. After binding of the primary antibody, cells were probed with a phycoerythrin conjugated goat anti-human IgG secondary antibody. Controls included cells only, VSTB124 and secondary antibody only.

For target validation of human VISTA in huVISTA knock-in mice, samples of spleen and tumour tissues of huVISTA knock-in as well as C57BL/6 control mice were used. To generate single cell suspensions tissues were finely minced, and digested for 45 min in Hanks Buffered Salt Solution (HBSS) containing 1 mg/mL collagenase type 4 (Worthington Biochemical Corp.) and 20 µg/mL DNase I (Roche) at 37 °C. Following digestion, tissues were passed through a 70-µm cell strainer and washed in PBS/1% FBS. Spleens were passed through a 70-µm cell strainer and treated with ammonium chloride potassium (ACK) buffer (0.15 M NH_4_Cl, 1 mM KHCO_3_, and 0.1 mM Na_2_ EDTA) to lyse erythrocytes and then washed in PBS/1% FBS. Single cell suspensions were FcR blocked using anti-CD16/32 (Biolegend) and then cell surface stained with VISTA AF647 (R&D Systems, FAB71261), CD45 PE (BD Biosciences), F480 FITC (eBioscience), CD11b Pacific Blue (Biolegend), TCRb APCCy7 (Biolegend), CD4 Pacific Blue (Biolegend), CD8 PeCy7 (Biolegend) and propidium iodide (Sigma). All stained cell suspensions were passed through a 40-µm filter before FACS acquisition.

All samples were run on a BD FACSCanto II (BD Biosciences) and data was analysed using FloJo v10 software.

### **Western blotting analysis of huVISTA target**

Protein lysates were prepared from cell pellets and tissues via lysis in RIPA buffer (10 mM Tris Tris-HCl pH 8.0, 1 mM EDTA, 1% TX-100, 0.1% sodium deoxycholate, 0.1% SDS, 150 mM NaCl). Lysates were quantitated using the Pierce BCA Protein Assay Kit (Thermo Fisher Scientific). 20 µg of protein was added to NU-PAGE SDS-PAGE loading dye with reducing agent (Thermo Fisher Scientific) and applied to 4–12% Bis-Tris CriterionTM XT gels (Thermo Fisher Scientific) run in MES buffer. Gels were transferred to activated PVDF (Thermo Fisher Scientific) using the iBlot2 gel transfer system (Thermo Fisher Scientific). Membranes were blocked in blocking buffer (3% skim milk in Tris Buffer Saline with 0.1% Tween 20 (TBST20)) for 1 h at room temperature. Membranes were probed with the following antibodies at 4 °C overnight: anti-huVISTA (Cell Signaling #64,953, 1:5000 in blocking buffer or Sigma #HPA007968, 1:1000 in blocking buffer) and anti-GAPDH (1:2000 in blocking buffer; Sigma G9545). Primary antibodies were detected with LI-COR IRDye 800CW anti-rabbit IgG secondary antibodies (LiCOR; 926-32211).

### Conjugation of deferoxamine-p-SCN to CI-8993 and control IgG1

CI-8993 (160 mg, 3.2 mL, 50 mg/mL) was buffer exchanged into sodium bicarbonate (0.1 M, pH 8.5) by PD-10 gel filtration as per the manufacturer’s instructions. The concentration of CI-8993 (152.2 mg) in sodium bicarbonate was adjusted to 10 mg/mL and a solution of Df-NCS (3.82 mg, 477 µL, 8 mg/mL) in DMSO was added at 5-fold molar excess (final DMSO concentration < 4%). The reaction mixture was incubated at ambient temperature (23 °C) for 1 h followed by PD-10 gel filtration using formulation buffer (20 mM sodium succinate pH 5.2, 8.5% sucrose, 0.04% Tween 20). The concentration of formulated Df-CI-8993 was adjusted to 6 mg/mL, the solution was sterile filtered, aliquoted, and stored at -80 °C.

Conjugation of human IgG1 (huIgG1) isotype control antibody was performed following the above procedure.

### Radiolabelling of CI-8993 and control human IgG1

A solution of [^89^Zr]Zr-oxalate (0.05 M, 15–130 MBq, 5-150 µL) was neutralised with an aqueous solution of sodium carbonate (0.1 M, pH 10.8) and diluted with formulation buffer (20mM sodium succinate pH 5.2, 8.5% sucrose, 0.04% Tween 20). Df-CI-8993 or Df-huIgG1 control in formulation buffer (4 µg/MBq) was added to neutralised ^89^Zr and the reaction mixture was incubated at ambient temperature (23 °C) for 15 min. Following incubation, purification was performed by PD-minitrap gel filtration (Cytiva) using formulation buffer as per the manufacturer’s instructions. Quality control was performed on purified radioconjugates with regards to radiochemical purity, protein integrity and serum stability as described previously [20]. [^89^Zr]Zr-Df-CI-8993 or [^89^Zr]Zr-Df-IgG1 control (20 µg) were incubated in healthy human donor serum (100 µL) at 37 °C and radiochemical purity was determined by iTLC and radio-SEC-HPLC over a period of 7 days.

### Recombinant human VISTA binding analyses with [^89^Zr]Zr-Df-CI-8993

Recombinant histidine-tagged extracellular domain of human VISTA/B7-H5/PD-1 H (rhVISTA; 19 kDa) was purchased from R&D Systems (9057-B7) for use in ELISA antigen binding analyses. ELISA 96 well Maxisorp plates (NUNC) were coated with rhVISTA antigen at 4 ºC overnight. Then, non-specific binding was blocked with 3% FCS-PBS (200 µL) at room temperature (RT) for 1 h. Serial titrations (15 µg/mL to 0.0146484 µg/mL) of CI-8993, Df-CI-8993, [^89^Zr]Zr-Df-CI-8993, or negative control huIgG1 isotype from at 50 µL/well, were added in duplicate and incubated at RT for 1 h. Secondary anti-huIgG Fc-HRP (Sigma A-0170) were applied (1:2000 dilution in 3%FCS-PBS) at 50 µL/well at RT for 1 h. Substrate 3,3 × 5,5´-tetramethylbenzidine (TMB, Sigma) was added at 50 µL/well and plates were incubated at RT for 10 min followed by 1M H_2_SO_4_ (50 µL /well) to stop the reaction. TMB colour was measured in an ELISA plate reader at 450 nm. Binding affinities were calculated using Prism (v10) using a log-log or 4-parameter curve fit.

### Animal model

In vivo investigations were performed in two preclinical models of VISTA expression. The first model utilized female humanized VISTA knock-in mice, C57BL/6 N-Vsir^tm1.1(VSIR)Geno^ (huVISTA knock-in mice), obtained from GenOway (Lyon, France) and purchased through Charles River Laboratories (Wilmington, MA, USA). Via a knock-in at the mouse VISTA locus, these mice express a chimeric VISTA molecule comprising a human extracellular, a human transmembrane and a murine intracellular domain [14]. Age matched female control C57BL/6 mice (8-week-old) were obtained from the Walter and Eliza Hall Institute of Medical Research (VIC, Australia). To establish syngeneic MB49 mouse bladder tumours, MB49 cells (0.3 × 10^6^ cells) were injected subcutaneously in C57BL/6 or huVISTA knock-in mice.

The second model involved a human xenograft tumour model using VISTA expressing pancreatic carcinoma cells. Capan-2 human pancreatic cancer cells (5 × 10^6^ cells in 50% matrigel) were injected subcutaneously in female BALB/c *nu*/*nu* mice (5–6-week-old) (Animal Research Centre, WA, Australia).

Tumour volume (TV) was calculated by the formula [(length × width^2^)/2] where length was the longest axis and width the measurement at right angles to length.

### Animal imaging and biodistribution studies in huVISTA knock-in and C57BL/6 mice

On Day 0, female C57BL/6 N-Vsir^tm1.1(VSIR)Geno^ mice (huVISTA KI) bearing established MB49 tumours (tumour volume, 79.24 ± 8.77 mm^3^) were injected with radiolabelled antibody via tail vein injection. Eight huVISTA KI mice received 100µL of 4.6MBq (124.7 µCi/24.6 µg; 1 mg/kg) [^89^Zr]Zr-Df-CI-8993 and eight huVISTA KI mice received 100µL of 4.6MBq (124.7 µCi/24.6 µg; 1 mg/kg) with 665.4 µg competing unlabelled CI-8993 (30 mg/kg) in formulation buffer (20mM succinate, 8.5% sucrose, 0.04% Tween 20 at pH 5.2). Control groups of four huVISTA KI mice and four C57BL/6 mice received 4.95 MBq (133.8 µCi/21.6 µg; 1 mg/kg) [^89^Zr]Zr-Df-IgG1 control antibody. In a separate control study, [^89^Zr]Zr-Df-CI-8993 antibody distribution was evaluated in C57BL/6 wild-type mice bearing established MB49 tumours (tumour volume, 75.68 ± 8.04 mm^3^): five C57BL/6 mice received 4.6MBq (124.8 µCi/20.66 µg; 1 mg/kg) [^89^Zr]Zr-Df-CI-8993 and five C57BL/6 mice received 4.6MBq (124.7 µCi/24.6 µg; 1 mg/kg) [^89^Zr]Zr-Df-CI-8993 with 668.89 µg competing unlabelled CI-8993 (30 mg/kg).

Two mice of each group were imaged with positron emission tomography (PET) and magnetic resonance (MR) on day 0 (2 h), day 1 and day 3 post injection using a dedicated small animal nanoPET/MR camera (Mediso, Hungary). After imaging, all mice were used for ex vivo biodistribution analysis on day 3 post injection. In addition, separate groups of four huVISTA KI mice injected with [^89^Zr]Zr-Df-CI-8993 (1 mg/kg as well as 30 mg/kg dose) were culled on day 1 post injection for ex vivo biodistribution analysis. Tissues, tumour and blood of all mice were collected and counted for radioactivity in a dual channel gamma scintillation counter (Wizard, PerkinElmer, Australia). Triplicate standards prepared from the injected material were counted at each time point with tissue and tumour samples enabling calculations to be corrected for the physical decay of the isotopes. The ex vivo biodistribution data were calculated as the mean ± SD percent injected dose per gram tissue (%ID/g) for each radiolabelled construct per time point.

Two huVISTA KI mice and two C57BL/6 wild-type mice bearing MB49 were used to collect tissues for western blot and FACS analysis to validate the presence and absence of huVISTA target expression.

To estimate PK of ^89^Zr-CI-8993 in the huVISTA model at the 1 mg/kg and 30 mg/kg dose we fitted a one-compartment model to the blood values obtained from 24 h to 72 h time points. Percentage injected dose per gram (%ID/g) was converted to µg/mL blood using injected dose of 24.6 µg and 690 µg. The Y-intercept at time of injection was estimated based on a total blood volume of 1.05 mL for the huVISTA KI mice (total blood volume is assumed 58.5 mL/kg per mouse and average body weight of the huVISTA mice was 17.88 g) [21]. Phoenix WinNonlin software was used to fit a one-compartment model.

### Animal imaging and biodistribution studies in Capan-2 tumour-bearing mice

On the day of injection, female BALB/c *nu*/*nu* mice bearing established Capan-2 tumours (tumour volume, 109.70 ± 36.28 mm^3^) were injected with 100 µL radiolabelled antibody via tail vein injection. Groups of three mice received 4.95MBq (133.8 µCi/22.5 µg; 1.3 mg/kg) [^89^Zr]Zr-Df-CI-8993, 4.95MBq (133.8 µCi/22.5 µg) with 147.5 µg competing unlabelled CI-8993 (total protein dose, 10 mg/kg) added or 4.95MBq (133.8 µCi/22.5 µg) with 487.5 µg competing unlabelled CI-8993 (total protein dose, 30 mg/kg) added.

Two mice of each group were imaged with positron emission tomography (PET) and magnetic resonance (MR) on day 0 (2 h), day 3 and day 7 post injection using a dedicated small animal nanoPET/MR camera (Mediso, Hungary). After imaging, all mice were used for ex vivo biodistribution analysis on day 7 post injection. The ex vivo biodistribution data were calculated as described above. Two Capan-2 bearing mice of each protein dose group were used to collect tissues for western blot analysis of huVISTA target expression.

### Statistical analysis

For comparisons of two groups, an unpaired t-test was used. For multiple comparisons, Brown-Forsythe one-way ANOVA was used, followed by a Dunnett’s T3 multiple comparisons test. All analyses were performed using Graphpad Prism version 10.03. Data are presented as the mean ± SD, unless stated differently.

## RESULTS

### Conjugation, radiolabelling and quality control of [^89^Zr]Zr-Df-CI-8993

**Fig. 1:**
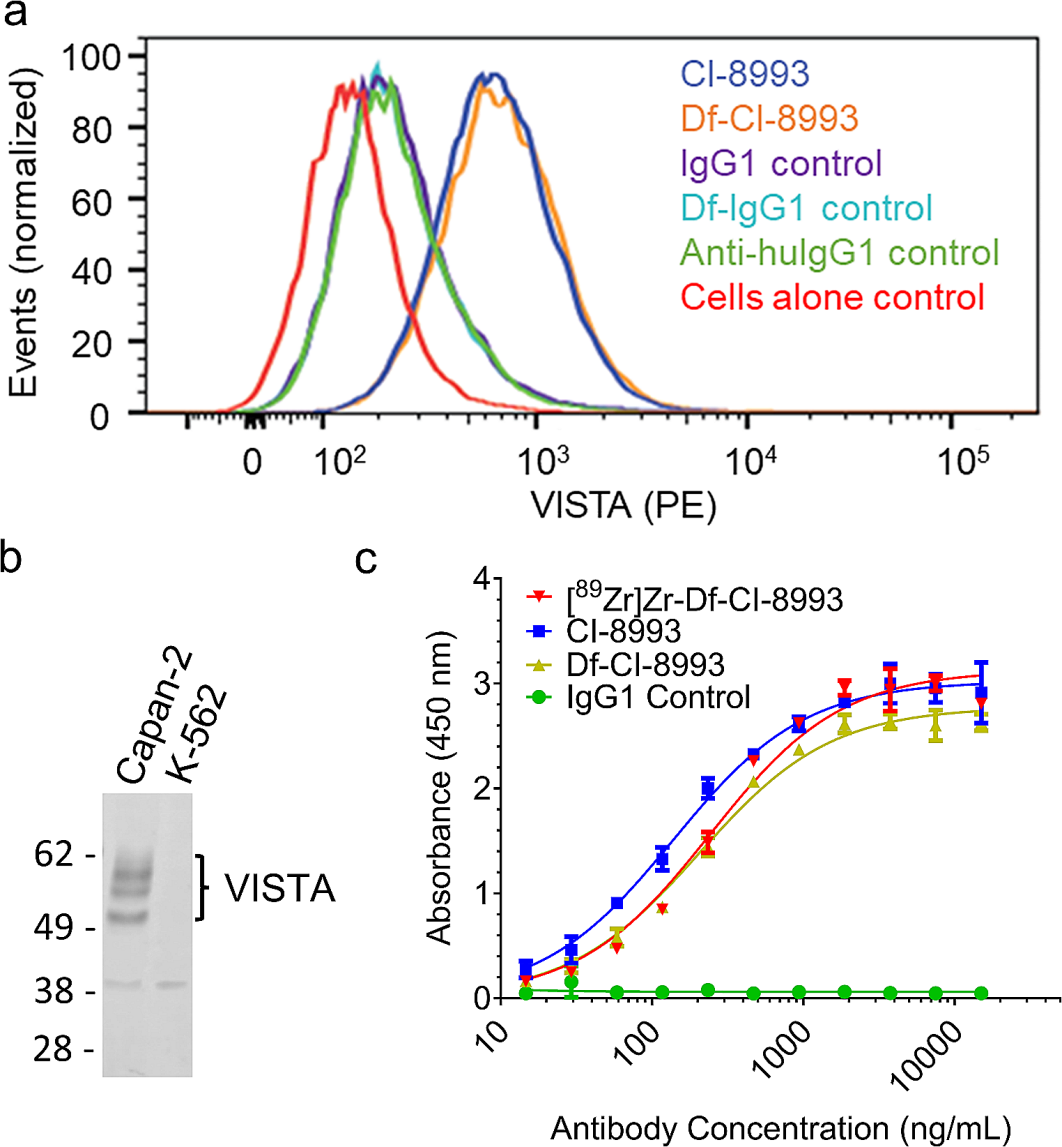
Binding analysis of [^89^Zr]Zr-Df-CI-8993, Df-CI-8993 and Df-controls. **a** Flow cytometry analysis showing binding of Df-CI-8993 and CI-8893 to human VISTA on human Capan-2 cells. **b** Western blot analysis confirming target expression of human VISTA in Capan-2 cells. K-562 cells show no expression of human VISTA. **c** ELISA assessment showing binding of CI-8993 to human VISTA was maintained after radiolabelling with ^89^Zr. Bars, mean ± SD; *n* = 3

Conjugation of Df-NCS to CI-8993 or isotype IgG1 control resulted in stable constructs with an average chelator-to-antibody ratio of 1.81-to-1 (Supplementary Fig. S1) and 2.11-to-1 (Supplementary Fig. S2), respectively. SDS-PAGE showed that the integrity of Df-conjugated antibodies was maintained after conjugation (Supplementary Fig. S3). FACS analysis with VISTA-expressing Capan-2 cells shows that binding of CI-8993 to VISTA was maintained after conjugation (Fig. 1a-b).

After radiolabelling, radiochemical purity was 94.31 ± 1.81% (*n* = 3) as determined via SE-HPLC (Supplementary Fig. S4) and 99.05 ± 0.41% (*n* = 3) via iTLC. Stability of radioimmunoconjugates was assessed in healthy human donor serum at 37 °C showing high retention of radiochemical purity over 7 days (Supplementary Table S1). Because none on the cell lines available showed high enough expression of human VISTA to perform a cell-based immunoreactivity assay, an ELISA assay was developed to evaluate the impact of radiolabelling on the immunoreactivity of CI-8993. Figure 1c shows there was no impact of conjugation or ^89^Zr-radiolabelling on binding of CI-8993 to human VISTA. Affinity (K_d_) was determined via non-linear regression assuming one-site binding (Graphpad prism) and was comparable for all three constructs ([^89^Zr]Zr-Df-CI-8993: 268.3 ± 36.6 nM; Df-CI-8993: 237.8 ± 44.1 nM; CI-8993: 298.9 ± 226.7 nM; *n* = 2).

#### In vivo imaging and biodistribution of [^89^Zr]Zr-Df-CI-8993 in female huVISTA knock-in mice

**Fig. 2:**
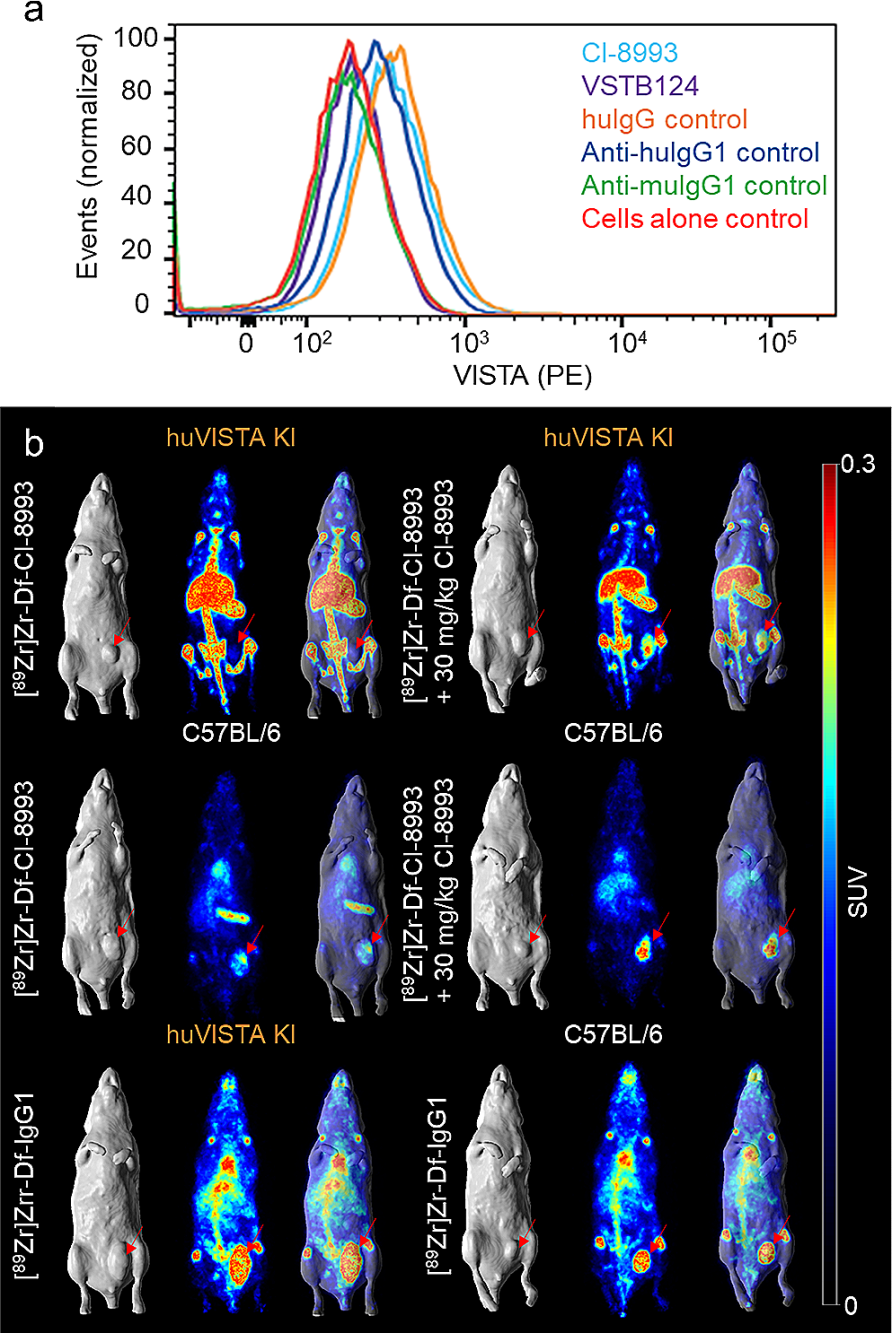
PET/MRI imaging of [^89^Zr]Zr-Df-CI-8993 in MB49-tumour-bearing huVISTA knock-in mice. **a** Flow cytometry analysis showing no binding of Df-CI-8993 and CI-8893 to MB49 murine bladder cancer cells. Controls include a murine anti-human VISTA binding antibody (VSTB124), a non-specific human IgG1 control antibody and secondary control antibodies (anti-murine IgG1 and anti-human IgG1). **b** From left to right, each panel shows a representative whole-body MR image (MRI, surface rendered), a maximum intensity projection PET image, and a fused PET/MRI images of MB49 tumour-bearing huVISTA knock-in mice or control C57BL/6 mice on day 3 post injection. Mice were injected with 1 mg/kg [^89^Zr]Zr-Df-CI-8993, 1 mg/kg [^89^Zr]Zr-Df-CI-8993 with 30 mg/kg unlabelled CI-8993, or [^89^Zr]Zr-Df-IgG1 control

The in vivo biodistribution and imaging properties of [^89^Zr]Zr-Df-CI-8993 and IgG1 isotype control radioconjugates were assessed in female huVISTA knock-in mice (C57BL/6 N-Vsir^tm1.1(VSIR)Geno^), expressing chimeric VISTA (extracellular and transmembrane human VISTA linked to intracellular murine VISTA domain) on their immune cells (CD4^+^ T cells, CD8^+^ T cells, Tregs and dendritic cells). The huVISTA knock-in mouse allows binding of the extracellular domain of chimeric VISTA by the CI-8993 antibody, whilst maintaining the intracellular function of VISTA in mice. C57BL/6 mice expressing murine VISTA were used as a negative control group. All mice were injected with murine MB49 bladder cancer cells. MB49 tumour cells do not express VISTA (Fig. 2a) but express the male H-Y antigen, which acts as a foreign antigen when injected into female mice. Under these conditions MB49 is a highly immunogenic tumour and will elicit an immune response.

Whole-body animal PET/MRI imaging and biodistribution was performed on day 1 and day 3. Table 1 shows the biodistribution results of [^89^Zr]Zr-Df-CI-8993 at two protein dose levels: 1 mg/kg and 30 mg/kg. Two mice of each group were imaged with positron emission tomography (PET) and magnetic resonance (MR). Figure 2b shows representative whole-body surface-rendered MR images, maximum intensity projection PET images, and fused PET/MRI images on day 3 post injection. Day 1 images are shown in supplementary figure S5.

**Table 1:** Biodistribution results of two protein doses of ^89^Zr-labelled CI-8993 in huVISTA knock-in mice on day 1 and day 3 post injection

	Uptake (%ID/g)*			
Tissues	Day 1		Day 3	
	1 mg/kg	30 mg/kg	1 mg/kg	30 mg/kg
	***(n*** **= 5)**	***(n*** **= 3)**	***(n*** **= 3)**	***(n*** **= 5)**
Blood	9.99 ± 0.73	5.69 ± 0.29	4.22 ± 0.19	0.42 ± 0.05
Brain	0.29 ± 0.05	0.28 ± 0.07	0.17 ± 0.05	0.12 ± 0.02
Heart	2.43 ± 0.27	3.29 ± 0.12	1.21 ± 0.14	1.93 ± 0.22
Lung	18.12 ± 0.82	6.73 ± 0.04	11.08 ± 0.41	4.07 ± 0.44
Stomach	1.30 ± 0.22	2.91 ± 0.14	0.69 ± 0.08	1.89 ± 0.15
Spleen	158.50 ± 24.00	37.15 ± 0.96	291.96 ± 20.53	41.15 ± 6.00
Liver	14.40 ± 1.64	8.09 ± 2.14	14.89 ± 0.70	8.14 ± 0.99
Kidney	5.95 ± 0.91	5.23 ± 0.46	5.57 ± 0.44	4.70 ± 0.47
Small Intestine	1.80 ± 0.35	3.62 ± 0.17	0.82 ± 0.10	2.77 ± 0.15
Colon	1.95 ± 0.34	3.49 ± 0.79	0.89 ± 0.03	2.56 ± 0.25
Muscle	0.85 ± 0.11	1.01 ± 0.04	0.75 ± 0.25	0.67 ± 0.09
Bone	10.88 ± 3.40	7.15 ± 0.53	13.44 ± 1.47	8.95 ± 0.96
Skin	1.34 ± 0.31	3.71 ± 0.39	0.84 ± 0.13	2.23 ± 0.43
Tail	3.18 ± 1.63	1.74 ± 0.07	2.21 ± 0.34	1.66 ± 0.15
Tumour	3.97 ± 0.68	12.47 ± 1.04	4.11 ± 0.84	8.30 ± 1.59

*Data presented as mean ± SD

In huVISTA knock-in mice, at the 1 mg/kg dose, both PET and biodistribution results showed high uptake of ^89^Zr-labelled CI-8993 in organs containing myeloid cells such as spleen (day 1, 158.50 ± 24.00%ID/g; day 3, 291.96 ± 20.53%ID/g) and bone (day 1, 10.88 ± 3.40%ID/g; day 3, 13.44 ± 1.47%ID/g), but at this dose level, the tumour uptake was low (day 1, 3.97 ± 0.68%ID/g, day 3, 4.11 ± 0.84%ID/g) (Fig. 2b; Table 1). In the presence of 30 mg/kg of unlabelled antibody, uptake in spleen and bone tissues were significantly reduced (day 1, 37.15 ± 0.96%ID/g and 7.15 ± 0.53%ID/g respectively; day 3, 41.15 ± 6.00%ID/g and 8.95 ± 0.96%ID/g respectively) and as a result, the tumour uptake was increased (8.30 ± 1.59%ID/g) demonstrating the specific uptake of ^89^Zr-Df-CI-8993 in MB49 tumour tissue.

In wild-type mice, uptake of [^89^Zr]Zr-Df-CI-8993 was significantly lower in spleen (12.55 ± 3.48%ID/g, *P* < 0.0001) and bone (2.68 ± 0.60, *P* < 0.0001) tissues on day 3 post injection (Fig. 2b; Table 2). In the presence of 30 mg/kg unlabelled CI-8993 antibody, uptake in the spleen of C57BL/6 mice expressing murine VISTA, was reduced to 4.66 ± 0.43%ID/g, suggesting some interaction between CI-8993 and splenocytes.

**Table 2:** Biodistribution results of two protein doses of ^89^Zr-labelled CI-8993 in C57BL/6 control mice on day 3 post injection

Tissues	Uptake (%ID/g)*	
	1 mg/kg	30 mg/kg
Blood	8.80 ± 1.00	7.08 ± 0.75
Brain	0.19 ± 0.03	0.16 ± 0.02
Heart	1.89 ± 0.13	1.88 ± 0.33
Lung	4.00 ± 0.42	3.29 ± 0.36
Stomach	1.01 ± 0.18	0.93 ± 0.10
Spleen	12.55 ± 3.48	4.66 ± 0.43
Liver	3.15 ± 0.64	3.50 ± 0.60
Kidney	4.05 ± 0.42	3.84 ± 0.42
Small Intestine	0.80 ± 0.13	0.74 ± 0.09
Colon	0.87 ± 0.14	0.84 ± 0.23
Muscle	0.57 ± 0.13	0.52 ± 0.06
Bone	2.68 ± 0.60	1.98 ± 0.24
Skin	0.96 ± 0.37	1.28 ± 0.16
Tail	1.05 ± 0.16	1.02 ± 0.13
Tumour	6.52 ± 1.30	8.50 ± 1.69

*Data presented as mean ± SD (*n* = 5)

Imaging and biodistribution results with ^89^Zr-labelled humanized IgG1 isotype control antibody did not show high uptake in myeloid rich organs, and as a result, higher blood pool activity was observed. As expected, there was no difference of tissue distribution of ^89^Zr-labelled IgG1 control antibody in huVISTA knock-in mice versus C57BL/6 mice (Fig. 2b; Table 3).

**Table 3:** Biodistribution results of ^89^Zr-labelled IgG1 control antibody at 1 mg/kg protein dose in in huVISTA knock-in mice and C57BL/6 control mice on day 3 post injection

Tissues	Uptake (%ID/g)*	
	huVISTA	C57BL/6
Blood	10.25 ± 0.24	12.66 ± 0.11
Brain	0.29 ± 0.04	0.36 ± 0.06
Heart	3.69 ± 0.35	4.21 ± 0.24
Lung	5.97 ± 0.30	7.64 ± 0.43
Stomach	2.34 ± 0.28	2.76 ± 0.29
Spleen	6.37 ± 0.38	7.18 ± 0.62
Liver	4.62 ± 0.26	4.89 ± 1.13
Kidney	5.52 ± 0.21	6.64 ± 0.57
Small Intestine	1.77 ± 0.24	2.05 ± 0.20
Colon	2.17 ± 0.14	2.26 ± 0.28
Muscle	1.30 ± 0.05	1.44 ± 0.02
Bone	6.43 ± 0.76	8.38 ± 1.57
Skin	4.20 ± 0.17	4.35 ± 0.12
Tail	2.40 ± 0.07	2.83 ± 0.44
Tumour	17.93 ± 1.92	21.29 ± 3.52

*Data presented as mean ± SD (*n* = 4)

To evaluate if [^89^Zr]Zr-Df-CI-8993 uptake in MB49 tumours in huVISTA knock-in mice was specific, tumour-to-blood ratios were calculated. Tumour-to-blood ratios indicated specific tumour uptake of [^89^Zr]Zr-Df-CI-8993 in the presence of 30 mg/kg unlabelled CI-8993 (20.47 ± 3.09) compared to 1 mg/kg [^89^Zr]Zr-Df-CI-8993 (0.97 ± 0.12; *P* = 0.0001) or ^89^Zr-Df-IgG1 control (1.75 ± 0.11; *P* < 0.0001). PK analysis of CI-8993 using ^89^Zr-Df-CI-8993%ID/g in blood at 1 mg/kg and 30 mg/kg are shown in Supplementary Table S2.

The expression of huVISTA in the huVISTA knock-in mice was confirmed via western blotting and FACS analysis using tissue samples collected from imaged mice (Suppl. Fig. S6a). We confirmed high expression of human VISTA in the spleen and expression in the MB49 tumour samples collected from huVISTA knock-in mice. As expected, no huVISTA was detected in tissue collected from C57BL/6 mice. FACS data showed that huVISTA knock-in mice expressed huVISTA in the spleen and on myeloid cells and some T cells (CD4 + and CD8+) infiltrating into the tumour (Suppl. Fig. S6b).

#### In vivo imaging and biodistribution of [^89^Zr]Zr-Df-CI-8993 in Capan-2 tumour-bearing BALB/c nude mice

**Fig. 3:**
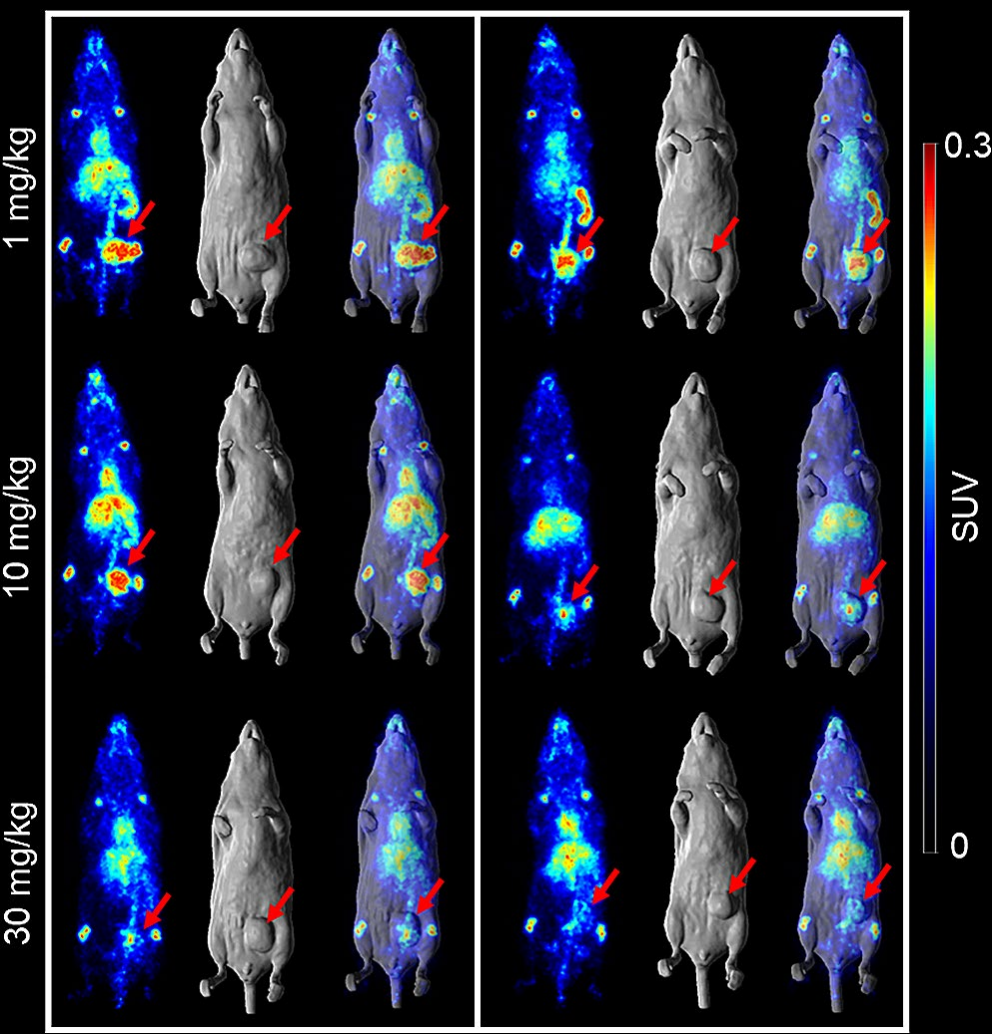
PET/MRI imaging of [^89^Zr]Zr-Df-CI-8993 in Capan-2 xenografted BALB/c nude mice. From left to right, each panel shows a representative whole-body MR image (MRI, surface rendered), a maximum intensity projection PET image, and a fused PET/MRI image. Capan-2 xenografted BALB/c nude mice were injected with 4.95 MBq (1.3 mg/kg [^89^Zr]Zr-Df-CI-8993) at three unlabelled protein doses: 1 mg/kg, 10 mg/kg and 30 mg/kg CI-8993. Two mice per protein dose were imaged on day 7 post injection

To evaluate the specific targeting of [^89^Zr]Zr-Df-CI-8993 in a VISTA-expressing human tumour model, we performed a second combined PET imaging and biodistribution study using Capan-2 xenografted BALB/c nude mice. Tissue distribution of [^89^Zr]Zr-Df-CI-8993 was evaluated at three different protein dose levels: 1 mg/kg, 10 mg/kg and 30 mg/kg unlabelled CI-8993. Figure 3 shows the imaging results obtained on day 7 post injection. At the lowest dose level, highest uptake was seen in spleen (1 mg/kg, 20.78 ± 4.02%ID/g; 10 mg/kg, 9.22 ± 1.04%ID/g, 30 mg/kg, 6.74 ± 0.57%ID/g) and tumour (1 mg/kg, 10.69 ± 4.02%ID/g; 10 mg/kg, 9.92 ± 3.19%ID/g, 30 mg/kg, 6.64 ± 0.57%ID/g) (Suppl. Fig S7a). PET images show some variability in heart and liver uptake of [^89^Zr]Zr-Df-CI-8993. This is in alignment with the variability seen in the individual mice used in the ex vivo biodistribution study as shown in supplementary Fig S7. For both spleen and tumour, uptake at 1 mg/kg was significantly higher than uptake at 30 mg/kg protein dose (*P* < 0.01). Increasing levels of competing unlabelled CI-8993, reduced the uptake in spleen and tumour, suggesting the specific targeting of CI-8993 in spleen and tumour in this mouse model. VISTA expression in Capan-2 tumours was confirmed via western blot (Suppl. Fig S8).

## Discussion

Although immune checkpoint blockade represents a promising approach in oncology exemplified by successful tumour responses observed with PD-1/PD-L1 and anti-CTLA-4 targeted therapies, careful patient selection and monitoring of the treatment response is mandatory due to the possible toxic side effects. Consequently, checkpoint-specific molecular imaging is being increasingly investigated as a tool for patient selection and response evaluation, and in novel immune targeted drug development [22]. In this study, we have developed an immunoPET tracer targeting immune checkpoint VISTA with the aim to evaluate and study the biodistribution and PK of CI-8893 in a human bioimaging study. CI-8993 maintained its structural integrity and immunoreactivity after conjugation and radiolabelling. Specific targeting of CI-8993 was mainly demonstrated in VISTA-expressing myeloid cells in spleen and blood. Specific uptake was also demonstrated in MB49 tumour tissue in a genetic mouse model expressing chimeric VISTA and in VISTA-expressing Capan-2 xenografts. Our results demonstrate that ^89^Zr-labelled CI-8993 is suitable for targeting and imaging VISTA expression in human trials.

To demonstrate VISTA target expression in our models, both western blotting and FACS analysis was used. Western blot analysis confirmed expression of VISTA in spleen and VISTA-expressing Capan-2 tumour tissue. However, western blotting was not sensitive enough to show a clear VISTA signal from tumour-infiltrating immune cells in the MB49 tumour samples, only picking up a low signal in MB49 tumour samples isolated from huVISTA mice. FACS analysis is more sensitive, and the technique allows enrichment for the immune cell population within the tumour. Using FACS, we could clearly demonstrate the presence of VISTA-expressing myeloid cells and leukocytes within MB49 tumour samples isolated from huVISTA mice but not in MB49 tumour samples isolated from C57BL/6 wildtype mice. Our findings support huVISTA expression and tissue distribution reported in previous preclinical studies that use this model for preclinical evaluation of huVISTA targeting antibodies [23, 24].

The first model utilized female humanized VISTA knock-in mice (C57BL/6 N-Vsir^tm1.1(VSIR)Geno^ (huVISTA knock-in mice)). Via a knock-in at the mouse VISTA locus, these mice express a chimeric VISTA molecule comprising a human extracellular, a human transmembrane and a murine intracellular domain to mimic the physiological regulation and expression pattern of the human VISTA [14]. In this model human VISTA is expressed on T regulatory, CD11b^High^ and CD4 and CD8 T cells. It is therefore expected that specific uptake of [^89^Zr]Zr-Df-CI-8993 will occur in organs containing lymphocytes. As a result, large differences were observed in spleen uptake and normal tissue distribution between the different models used. Whilst the huVISTA knock-in mice highly express huVISTA in the spleen, resulting in spleen uptake of 291.96 ± 20.53%ID/g on day 3 p.i., the wild-type C57BL/6 (day 3, 12.55 ± 3.48%ID/g) and BALB/c nude mice (day 7, 20.78 ± 4.02%ID/g) only expressing murine VISTA show less spleen uptake. Competition with 30 mg/kg unlabelled CI-8993 was not sufficient to fully block the spleen uptake in the huVISTA KI model, still showing 37.15%ID/g and 41.15%ID/g in the spleen on day 1 and day 3 respectively, which is significantly higher than the spleen uptake seen in the MB49 tumour-bearing wild-type C57BL/6 and Capan-2 tumour-bearing BALB/c nude mice. Consequently, in the huVISTA knock-in mice, as the huVISTA targets in the spleen get more saturated at the 30 mg/kg protein dose, more [^89^Zr]Zr-Df-CI-8993 becomes available to reach the tumour. Other tissues that showed a significant reduction in the presence of 30 mg/kg unlabelled CI-8993 were blood, liver, lung, and bone. All these VISTA target rich sites contribute to the antigen sink and need to be saturated to allow increase in MB49 tumour uptake. The absolute tumour uptake in the huVISTA tumour model therefore shows an increase from 3.97%ID/g to 12.47%ID/g on day 1, suggesting the protein dose for highest tumour uptake might not have been fully reached. We did not escalate the protein dose further, as this would not be necessarily translatable to human studies. In contrast, the 30 mg/kg is sufficient to reduce the lower spleen uptake observed in the Capan-2 tumour-bearing BALB/c nude mice, and therefore, in the Capan-2 tumour-bearing mice, a reduction in tumour uptake can be observed at 30 mg/kg.

Previous in vitro studies demonstrated binding of CI-8893 to human and cynomolgus monkey VISTA but not to murine VISTA (data not shown) and therefore it is unlikely that direct binding of CI-8993 to murine VISTA contributed to spleen uptake in the mouse models only expressing wild-type murine VISTA. It is possible Fc interactions from CI-8993 to Fcγ receptors contribute to the uptake in mouse spleen because human Fc tends to bind more strongly to murine Fcγ receptors compared to their murine counterparts [25]. Spleen cells are more accessible to CI-8993 compared to tumour cells and therefore Fcγ interactions will be reduced in the presence of increased unlabelled CI-8993. Other irreversible non-specific binding mechanisms contribute to uptake to uptake of ^89^Zr-antibodies in normal tissues [26]. Smilarily, in the huVISTA knock-in model, uptake in liver, bone and joints will be a combination of the presence of lymphocytes, Fcγ receptor interaction and free ^89^Zr uptake resulting from catabolism. Based on our in vitro stability data, there is no reason to conclude that the antibody is unstable. However, some free ^89^Zr over time is expected and has been reported by us and others as part of normal antibody catabolism and distribution properties of free ^89^Zr [25–32].

Similarly to other immune checkpoint targeting antibodies with target expression also available in non-tumour tissue (i.e. lymphoid tissue), the absolute MB49 tumour uptake in the huVISTA expressing mouse model is lower than seen with antibodies against many receptors expresed on tumour cells, but quite similar to that seen with antibodies to immune targets [24, 28]. Consistent with other immune checkpoints, VISTA expression in non-tumour tissue is higher and therefore CI-8993 in vivo performance characteristics are similar to radiolabelled PD-L1 targeting antibodies. Although we observed modest absolute tumour uptake of [^89^Zr]Zr-Df-CI-8993 in the genetic model, the purpose of this preclinical model was to confirm the specific binding properties of [^89^Zr]Zr-Df-CI-8993 to huVISTA in mouse tissues made to express huVISTA. Specific targeting of [^89^Zr]Zr-Df-CI-8993 was demonstrated in the huVISTA KI model by the reduction of [^89^Zr]Zr-Df-CI-8993 in lymphocyte rich organs (blood, spleen, liver, lung, bone) and by the increase in tumor-to-blood ratio from 0.97 at 1 mg/kg to 20.47 at 30 mg/kg antibody dose. In addition, uptake of ^89^Zr-labelled CI-8993 in C57BL/6 control mice is typical of non-specific uptake of an antibody in the tumour and is related to blood pool activity. Therefore, in the presence of 30 mg/kg unlabelled CI-8993, this uptake was not changed. Importantly, at the 30 mg/kg dose level the tumour: blood ratio of [^89^Zr]Zr-Df-CI-8993 was markedly increased, demonstrating the specificity for VISTA target when normal tissue expression was blocked. The amount of huVISTA expression as well as the accessibility of VISTA in this genetic model might be different in humans. A clinical trial study with [^89^Zr]Zr-Df-CI-8993 is more appropriate to investigate the availability of huVISTA in normal tissue in patients and how much unlabelled huVISTA would be sufficient to block huVISTA expression in normal tissue. Recent pharmacokinetic and pharmacodynamic data from a phase 1 study of CI-8993 anti-VISTA antibody in patients with advanced solid tumours showed safe delivery of anti-VISTA antibody up to a full dose of 0.6 mg/kg. Increased half-life at 0.6 mg/kg was observed compared to 0.15 mg/kg suggesting the ability to saturate VISTA in normal tissues. Results of 1, 2 and 4 mg/kg dose in patients have not been published yet [33]. This suggests that in contrast to the 30 mg/kg unlabelled CI-8993 needed to saturate huVISTA in the genetic mouse model, a dose of 1 mg/kg might be sufficient in humans.

An additional control to confirm the proportion of [^89^Zr]Zr-Df-CI-8993 taken up by the tumour due to the immune response could be a group of MB49-tumour bearing mice injected with 1 mg/kg [^89^Zr]Zr-Df-CI-8993 with or without 30 mg/kg unlabelled CI-8993, although this was beyond the scope of this manuscript. These male mice will not elicit an immune response to the H-Y antigen and may show non-specific uptake of [^89^Zr]Zr-Df-CI-8993 at both antibody levels.

Unlike PD-1 and CTLA-4, VISTA expression is prominent in myeloid cells, including neutrophils, tumour-infiltrating leukocytes and myeloid-derived suppressor cells (MDSCs), which play a critical role in suppressing tumour-specific T-cell responses [19, 34]. VISTA blockade has been shown to reduce MDSCs and neutrophils by mechanisms distinct from PD-1 and CTLA-4, both in preclinical models and in clinical trials [18]. Targeting VISTA has shown great promise in multiple preclinical models and can decrease myeloid suppression of immune responses as well as induce proinflammatory changes in the tumour microenvironment by mechanisms different from PD-1 and CTLA-4 blockade [19]. Therapeutics targeting VISTA have entered clinical trials and have the potential to markedly enhance the efficacy of conventional immunotherapy treatment of cancer patients [19].

## Conclusion

In conclusion, we have developed [^89^Zr]Zr-Df-CI-8993 for specific binding to huVISTA, and which can be used to image VISTA expression in tumour and normal tissues. This approach has direct relevance to the development of CI-8993 as a novel immuno-oncology drug in human trials.

## Electronic supplementary material

Below is the link to the electronic supplementary material.

Supplementary Material 1

## Data Availability

The datasets generated during and/or analysed during the current study are available from the corresponding author on reasonable request.
