# Correction to: Targeting of immune checkpoint regulator V-domain Ig suppressor of T-cell activation (VISTA) with ^89^Zr-labelled CI-8993

**DOI:** 10.1007/s00259-024-06869-6

**Published:** 2024-08-26

**Authors:** Ingrid Julienne Georgette Burvenich, Christian Werner Wichmann, Alexander Franklin McDonald, Nancy Guo, Angela Rigopoulos, Nhi Huynh, Mary Vail, Stacey Allen, Graeme Joseph O’Keefe, Fiona Elizabeth Scott, Raul Soikes, Steven Angelides, Reinhard von Roemeling, Andrew Mark Scott, Ingrid Julienne, Georgette Burvenich

**Affiliations:** 1 Tumour Targeting Laboratory, Olivia Newton-John Cancer Research Institute, Level 5 ONJ Centre, 145 Studley Road, Heidelberg, VIC 3084 Australia; 2 School of Cancer Medicine, La Trobe University, Melbourne, VIC Australia; 3 Curis Inc, Lexington, MA USA; 4 Department of Medicine, University of Melbourne, Melbourne, VIC Australia; 5 Department of Molecular Imaging and Therapy, Austin Health, Melbourne, VIC Australia

**Correction to: European Journal of Nuclear Medicine and Molecular Imaging**

10.1007/s00259-024-06854-z

The authors regret that the below graphical abstract was not captured in the original published article online.


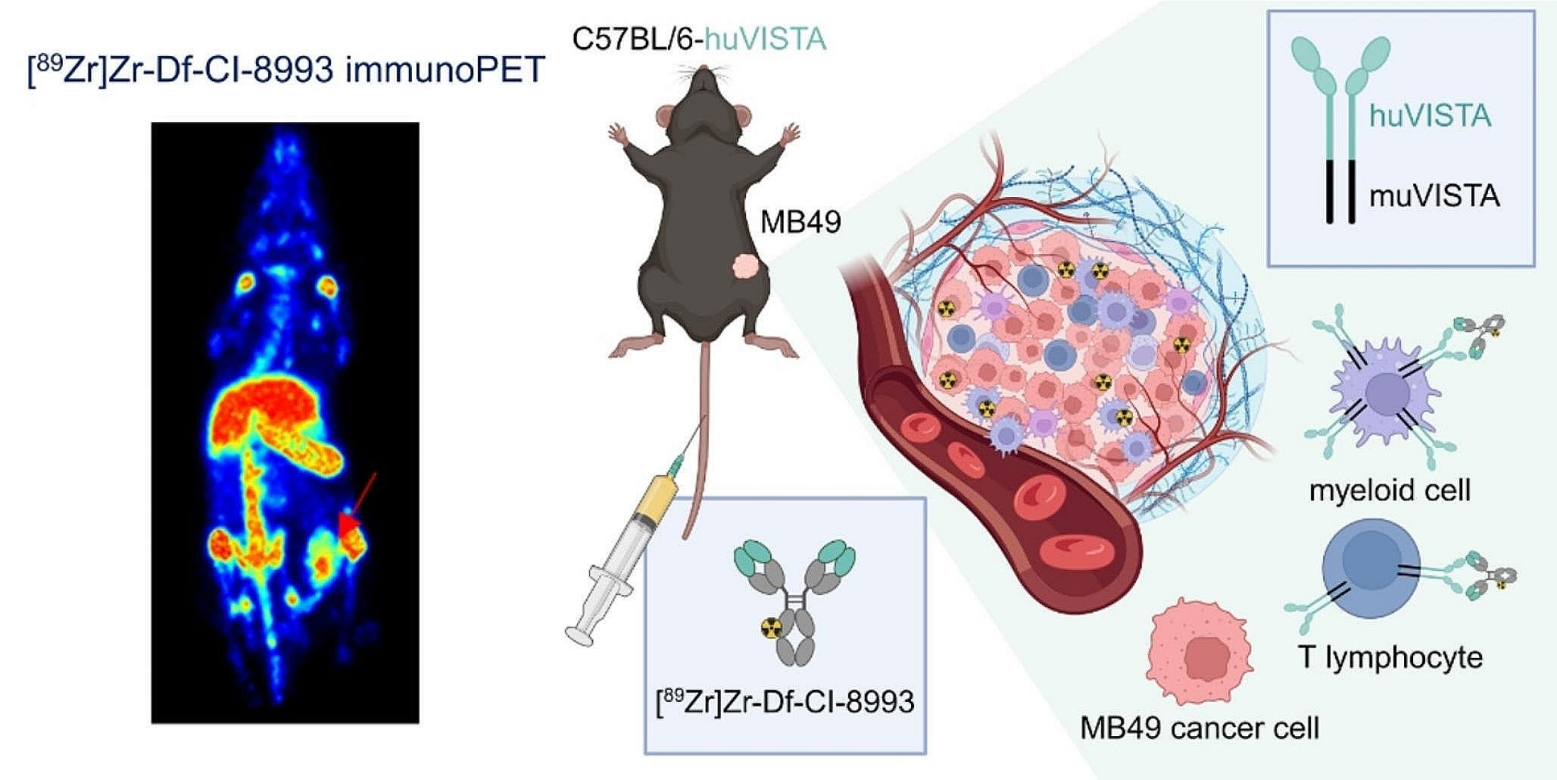


The original article has been corrected.

