# Supplementary material for: Targeting of immune checkpoint regulator V-domain Ig suppressor of T-cell activation (VISTA) with 89Zr-labelled CI-8993
Source: Eur J Nucl Med Mol Imaging. 2024 Jul 26;51(13):3863–73. doi: 10.1007/s00259-024-06854-z (PMC11527895; doi:10.1007/s00259-024-06854-z)
Supplement: Supplementary file 1 — Supplementary Material 1 [file 259_2024_6854_MOESM1_ESM.docx]

**Targeting of immune checkpoint regulator V-domain Ig suppressor of T-cell activation (VISTA) with ^89^Zr-labelled CI-8993**

Ingrid Julienne Georgette Burvenich^1,2*^, Christian Werner Wichmann^1,2*^, Alexander Franklin McDonald^1,5^, Nancy Guo^1^, Angela Rigopoulos^1^, Nhi Huynh^1^, Mary Vail^1,2^, Stacey Allen^1,2^, Graeme Joseph O’Keefe^5^, Fiona Elizabeth Scott^1^, Raul Soikes^3^, Steven Angelides^3^, Reinhard von Roemeling^3^, and Andrew Mark Scott^1,2,4,5^

^1^Tumour Targeting Laboratory, Olivia Newton-John Cancer Research Institute, Melbourne, VIC, Australia

^2^School of Cancer Medicine, La Trobe University, Melbourne, VIC, Australia

^3^Curis Inc, Lexington, MA, USA

^4^Department of Medicine, University of Melbourne, Melbourne, VIC, Australia

^5^Department of Molecular Imaging and Therapy, Austin Health, Melbourne, VIC, Australia

*authors contributed equally

**Corresponding Author:**

Professor Andrew M. Scott,

Tumour Targeting Laboratory, Olivia Newton-John Cancer Research Institute, Level 5 ONJ Centre, 145 Studley Road, Heidelberg, Victoria 3084, Australia

Phone: 61-39496-5876; Fax: 61-39496-5334;

E-mail: andrew.scott@onjcri.org.au

**First Authors:**

Ingrid Burvenich PhD (Senior Research Scientist, NIF Facility fellow) and Christian Wichmann (Senior Research Scientist), Tumour Targeting Laboratory, Olivia Newton-John Cancer Research Institute, Level 5 ONJ Centre, 145 Studley Road, Heidelberg, Victoria 3084, Australia

Phone: 61-39496-5876; Fax: 61-39496-5334;

Email: ingrid.burvenich@onjcri.org.au; Christian.wichmann@onjcri.org.au

**Short Title:** [^89^Zr]Zr-Df-CI-8993 imaging of immune checkpoint regulator VISTA

**INDEX**

**Supplementary Figures S1, S2, S3, S4, S5, S6, S7, S8.**

**Supplementary Table S1, Table S2.**

**
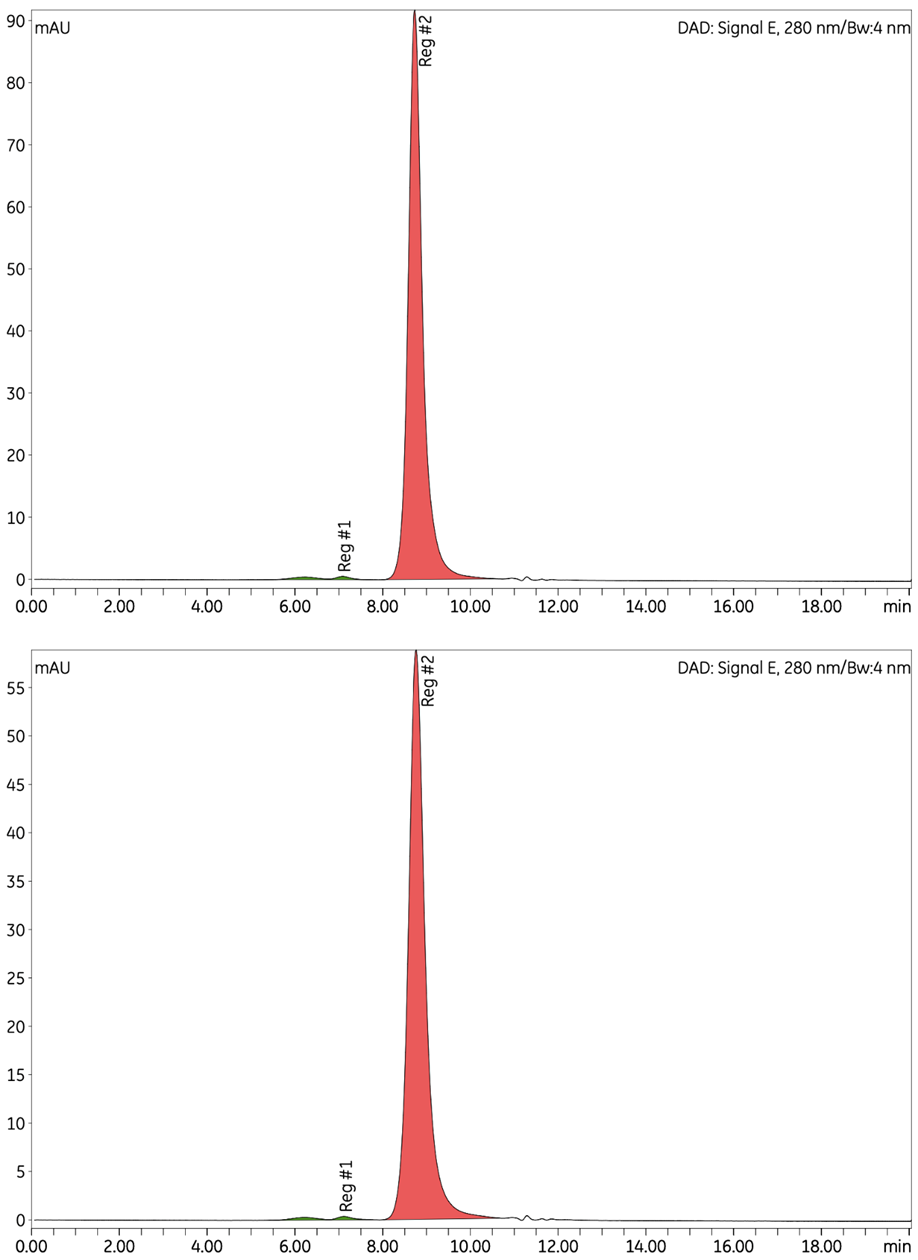
**

**Suppl. Fig. S1** SEC-HPLC analysis. A280 chromatograms of CI-8993 (top) and Df-conjugated CI-8993 (bottom) demonstrated retention of protein integrity.


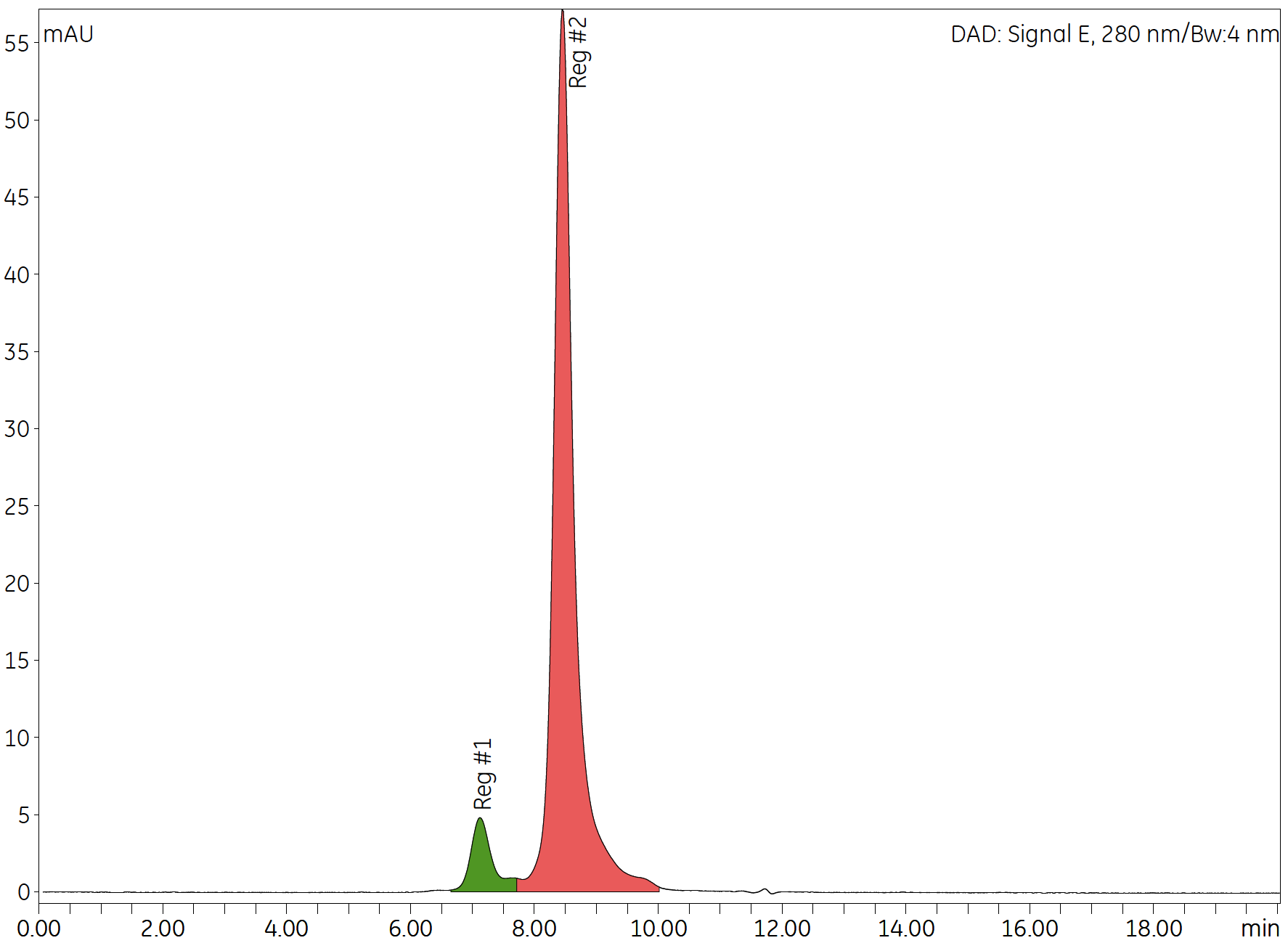


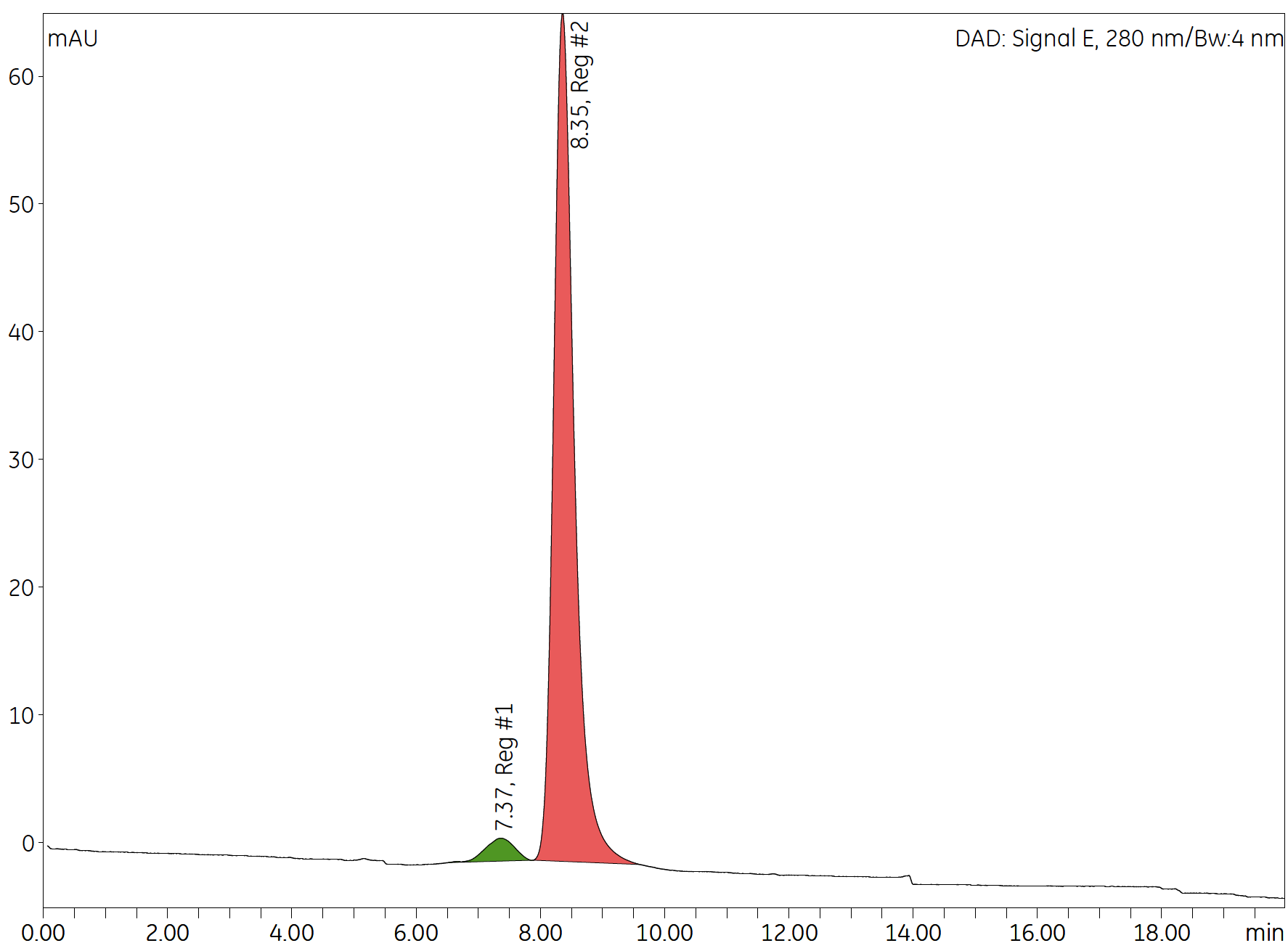


**Suppl. Fig. S2** SEC-HPLC analysis. A280 chromatograms of isotype control (top) and Df-conjugated isotype control (bottom) demonstrated retention of protein integrity.


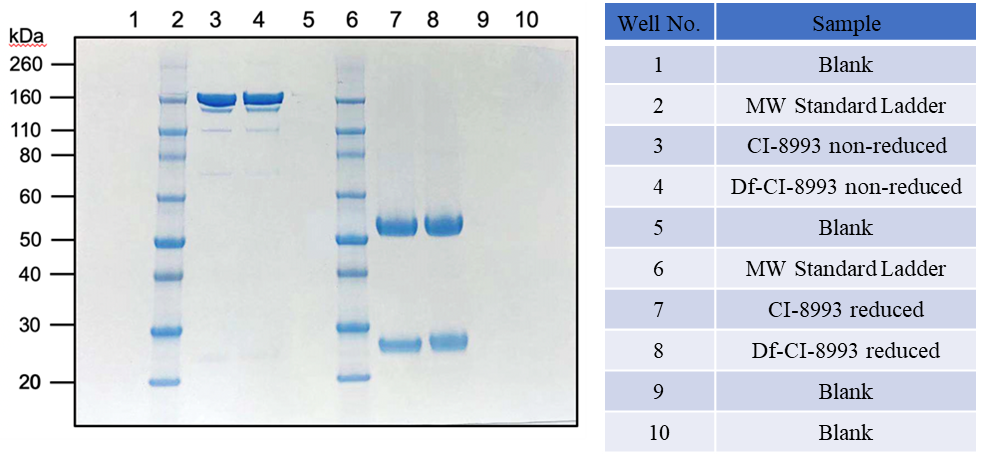


**Suppl. Fig. S3.** SDS-PAGE analyses of CI-8993 and Df-CI-8993 drug product samples (5 mg) under non-reduced and reduced conditions. The Df-CI-8993 was prepared without loss of fusion protein structural integrity.

**
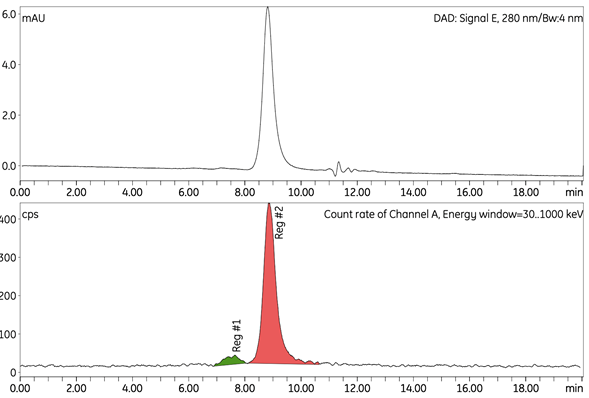
**

**Suppl. Fig. S4.** SEC**-**HPLC SEC analysis. A280 (top) and RAD (bottom) chromatograms of [^89^Zr]Zr-Df-CI-8993. Radiolabelled CI-8993 demonstrated retention of protein integrity and the retention time of the radiation monomer peak correlates with the A280 monomer peak.

**
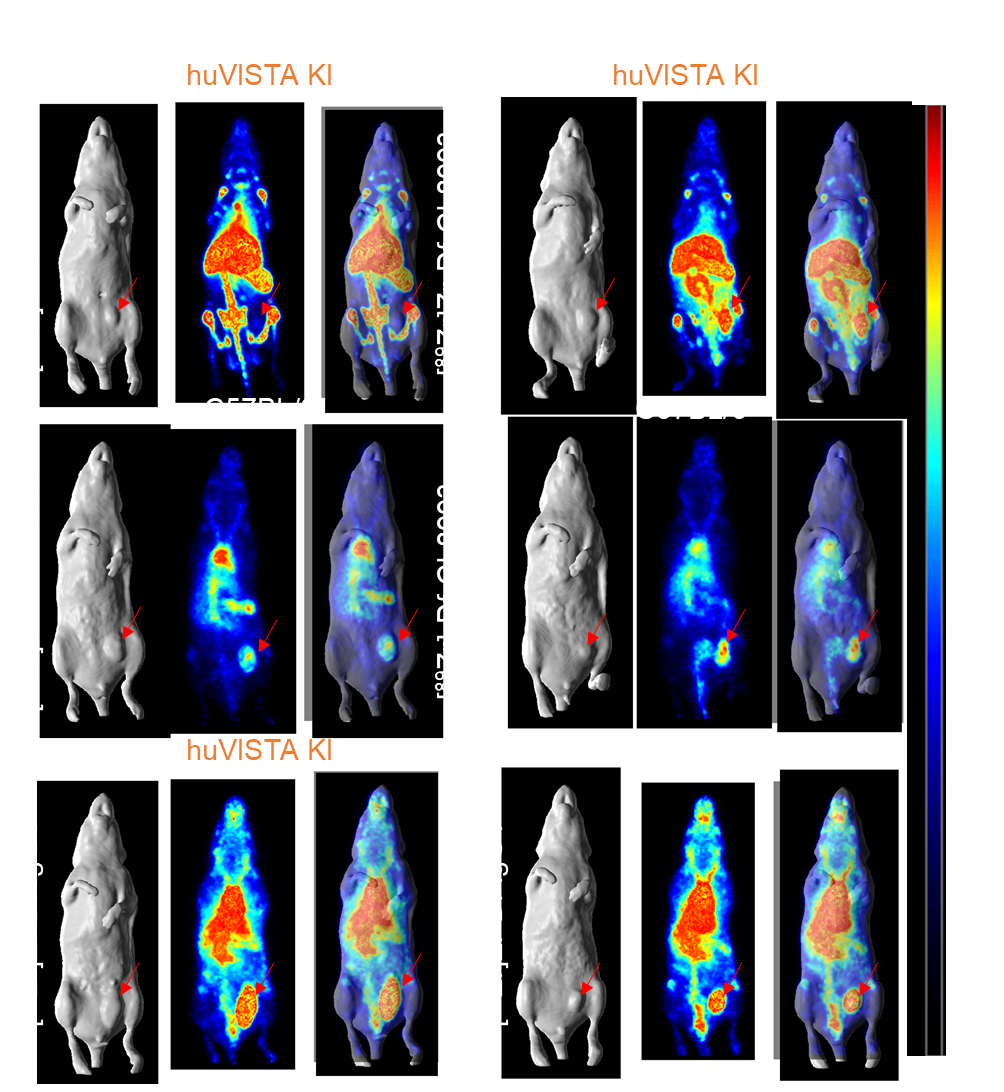
**

**Suppl. Fig. S5.** PET/MRI imaging of [^89^Zr]Zr-Df-CI-8993 in MB49-tumour-bearing huVISTA knock-in mice on day 1 post injection. From left to right, each panel shows a representative whole-body MR image (MRI, surface rendered), a maximum intensity projection PET image, and a fused PET/MRI image of MB49 tumour-bearing huVISTA knock-in mice or control C57BL/6 mice on day 1 post injection. Mice were injected with 1 mg/kg [^89^Zr]Zr-Df-CI-8993, 1 mg/kg [^89^Zr]Zr-Df-CI-8993 plus 30 mg/kg unlabelled CI-8993, or [^89^Zr]Zr-Df-IgG1 control.


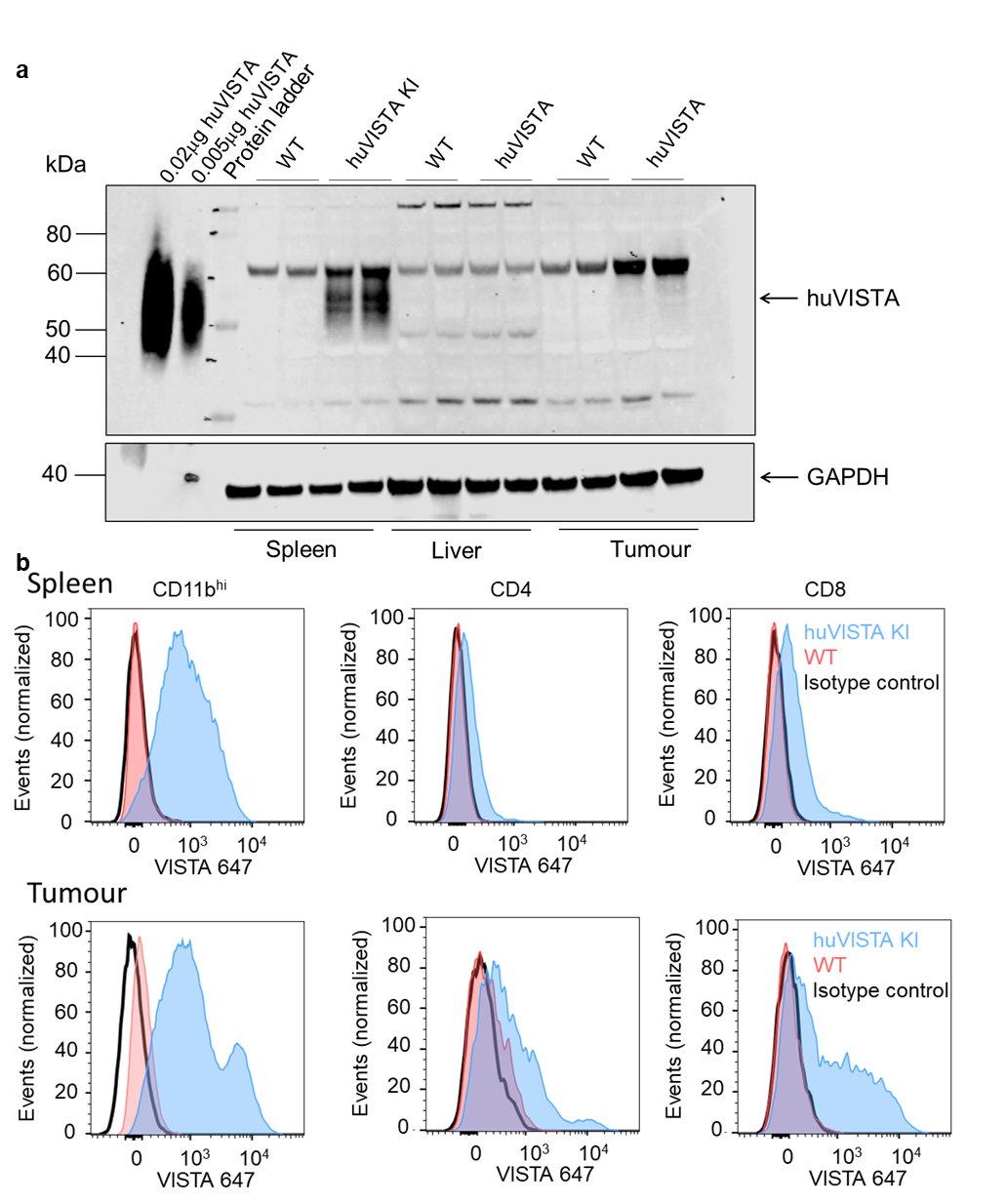


**Suppl Fig. S6.** huVISTA target validation in huVISTA knock-in mice used in imaging and biodistribution studies. **a** Western blot (anti-VISTA, Sigma #HPA007968) showing expression of the huVISTA target in two representative spleen and tumour samples collected from huVISTA knock-in (huVISTA KI) mice. huVISTA is not detected in samples collected from wild-type (WT) C57BL/6 mice. Recombinant human VISTA protein was used as a positive control. **b** FACS analysis showing huVISTA expression in myeloid and CD4+ and CD8+ T cell population of spleen and tumour samples collected from huVISTA KI mice (blue). As expected, no huVISTA was detected in samples collected from C57BL/6 mice (red).

**
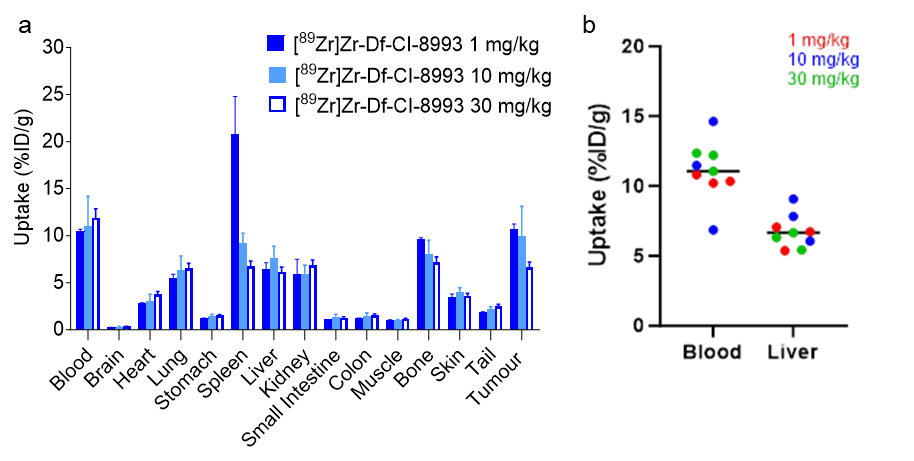
**

**Suppl Fig. S7**. Ex vivo uptake of [^89^Zr]Zr-Df-CI-8993 in Capan-2 tumour-bearing BALB/c nude mice. **a** Biodistribution results of [^89^Zr]Zr-Df-CI-8993 in Capan-2 xenografted BALB/c nude mice on day 7 post injection. *n* = 3; bars, SD **b** The individual data points measured for each group in the day 7 ex vivo biodistribution study for heart and liver are shown. Points in red show the data obtained from the 1 mg/kg dose group, points in blue are obtained from the 10 mg/kg dose, and the points in green are taken from the 30 mg/kg dose group. Blood values range from 6.85 %ID/g to 14.63 %ID/g. Liver uptake ranges between 5.37 %ID/g and 9.07 %ID/g. The variability in uptake of [^89^Zr]Zr-Df-CI-8993 in heart and liver seen in the individual PET images are aligned with the uptake range in blood and liver ex vivo.


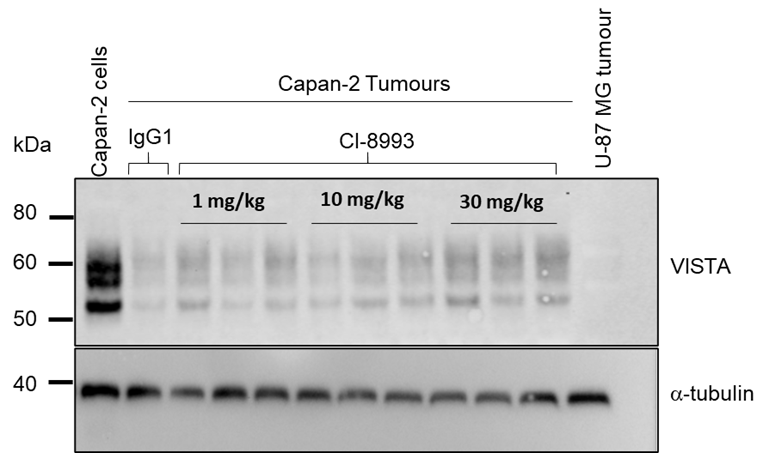


**Suppl Fig. S8.** Western blot (anti-VISTA, Cell Signaling #64953) showing expression of the huVISTA target in Capan-2 tumours collected from the biodistribution and imaging study using Capan-2-bearing BALB/c nude mice injected with different protein doses of [^89^Zr]Zr-Df-CI-8993 (1 mg/kg, 10 mg/kg, 30 mg/kg). Controls include Capan-2 cells, Capan-2 tumour injected with ^89^Zr-Df-IgG1 control and U-87 MG huVISTA-negative tumour. α-tubulin was used as a loading control.

**Suppl Table S1.** Stability analysis of radioimmunoconjugates in healthy human donor serum at 37°C.

| **Antibody** | **Method** | **Day 0** | **Day 2** | | **Day 7** | |
| --- | --- | --- | --- | --- | --- | --- |
| **[^89^Zr]Zr-Df-CI-8993 (n=3)** | iTLC | 99.05% ± 0.41% | 98.76% ± 0.19% | | 97.41% ± 1.21% | |
|  | SEC-HPLC | 94.31% ± 1.81% | 89.20% ± 2.30%^*^ | | 87.72% ± 2.21% | |
| **[^89^Zr]Zr-Df-IgG1 (n=1)** | iTLC | 99.71% | 99.46% | | 99.44% | |
|  | SEC-HPLC | 97.12% | 92.09% | | 84.13% | |
| ******Measurement of this sample was taken on day 3* | | | | | | |
|  |  |  | |  | |  |

**Suppl Table S2.** PK analysis following a single dose based on mean blood concentration of [^89^Zr]Zr-Df-CI-8993 at 24 h and 72 h post injection in hVISTA KI Mice

|  | [^89^Zr]Zr-Df-CI-8993 dose | |
| --- | --- | --- |
| Parameters | 1 mg/kg | 30 mg/kg |
| AUC (h ×μg/mL) | 251.03 ±45.86 | 5598.76 ±140.47 |
| t_1/2_ (h) | 7.43 ± 1.35 | 5.91 ±0.15 |
| C_max_ (μg/mL) | 23.43 ± 1.01 | 657.14 ±2.76 |
| C_L_ (mL/h) | 0.098 ±0.018 | 0.123 ±0.003 |
| Vss (mL) | 1.050 ±0.045 | 1.050 ±0.004 |
